# Prevalence and determinants of unmet need for contraception in North Gonja District, Ghana

**DOI:** 10.1186/s12905-020-01077-4

**Published:** 2020-10-06

**Authors:** Anthony Wemakor, Humphrey Garti, Nawaf Saeed, Obed Asumadu, Bede Anyoka

**Affiliations:** School of Allied Health Sciences, University for Development Studies, Tamale, Ghana

**Keywords:** Contraception, family planning, unmet need, North Gonja, Ghana

## Abstract

**Background:**

Unmet need for contraception contributes to the burden of unwanted pregnancies, which are correlated with a host of adverse maternal and child outcomes. The aim of this study was to determine the prevalence and identify the determinants of unmet need for contraception in North Gonja District, Ghana.

**Methods:**

A cross-sectional survey involving 386 randomly selected women of childbearing age was conducted in North Gonja district, Ghana, with the use of a questionnaire in household interviews. Women were classified as having unmet need for contraception if they were fecund, sexually active and wished to postpone the next birth or halt childbearing completely but were not using any form of contraception. Chi-square/Fisher’s exact test and logistic regression analysis were used to identify the determinants of unmet need.

**Results:**

The mean age of the study population was 26.1 (±8.4) years and awareness on contraception was almost universal in the district (95.9%). The overall prevalence of unmet need for contraception was 38.9%, with 27.5% having unmet need for limiting and 12.2% unmet need for spacing. In multivariate analysis, compared to women aged 25–29 years, those aged 20–24 years [Adjusted Odds Ratio (AOR) 0.26; 95% Confidence Interval (CI) 0.11–0.58] and 30 years and above (AOR 0.25; 95% CI 0.09–0.73) were less likely to have unmet need for contraception. However, uneducated women (AOR 5.06; 95% CI 1.07–24.01) compared with those educated to tertiary level; those unaware of family planning (AOR 3.93; 95% CI 1.12–13.80) compared to those aware; and those who had not previously practised contraception (AOR 1.81; 95% CI 1.09–3.00) compared to those who did were more likely to have unmet need.

**Conclusions:**

The present study found high prevalence of both awareness on and unmet need for contraception among the study population. Unmet need for contraception is associated with age, educational status, awareness on family planning and previous contraception practice. Educational campaigns to promote contraception should prioritize women of middle age and low educational status. Further studies are needed to understand the low correlation between awareness on and unmet need for contraception.

## Background

Pregnancy and childbirth are important causes of mortality among women of reproductive age, especially in low resource settings [1]. About half of all pregnancies are unplanned [2], which contributes significantly to maternal mortality burden [3]. Maternal health is an issue of concern globally and is highlighted in the third Sustainable Development Goal that aims to reduce maternal mortality ratio globally to not more than 70 maternal deaths per 100,000 live births by 2030 [4]. Unmet need for contraception is of great significance given that approximately 29% of maternal deaths could be prevented through met need for contraception [5]. Unmet need for contraception refers to the percentage of fecund (do not meet the criteria for infecundity) and sexually active women (married or in a union) of reproductive age who wish to postpone the next birth for at least 2 years or halt child bearing completely but are not using any form of contraception [6]. The 2012 London Summit on Family Planning (FP) called for increased investments in programmes to minimize unintended pregnancies worldwide and set a goal of satisfying the unmet need for FP of 150 million additional women by 2020 [7].

In Western Africa, only one in four women of reproductive age used a modern method of contraception in 2012 and modern contraceptive use was considerably low in many countries of the sub-region [8]. Sub-Saharan Africa had the highest level of unmet need for contraception in 2010 (25%). Out of 35 countries, 24 had unmet need prevalence rate of more than 20% and 7 of these had rates above 30% in 2011 [9]. The recent Ghana Demographic and Health Survey estimated the level of unmet need for contraception for married women in Ghana to be 30% but it varies with age, region of residence, educational status and household wealth index [10]. Unmet need is highest among teenagers (15–19 years) (50.7%) but lowest in older women (45–49 years) (14.2%), comparatively higher in Volta and Eastern Regions (above 35.0%), and lowest in women with at least secondary education (24.1%) or those in the richest wealth quintile (25.3%). Some of the reasons why women do not use a contraceptive method are: lack of knowledge on contraceptive usage [11], concerns about health [11], side effects of contraceptives [12], behavioural requirements and objections from husbands [13].

In Ghana, health care delivery is provided by both public and private sectors with the Ministry of Health having oversight responsibility over the whole health system. The country has a three-tier decentralized health system consisting of district (primary), regional (secondary), and national (tertiary) levels. Health services are provided largely by Ghana Health Service, Teaching Hospitals and faith-based, quasi- and non-governmental health facilities [14]. All forms of modern contraceptives (IUDs, implants, injectables, oral contraceptive pills and condoms) are distributed at highly subsidized rates by hospitals, clinics, health centres and community-based health planning and services compounds. Other distribution outlets are pharmacies, over the counter medicine sellers and community health volunteers for rural communities.

The prevalence of unmet need for FP is highest in low and middle income countries including Ghana [15] but very little is known about its determinants. Similarly, there is very scanty data on the level of unmet need and its determinants in North Gonja District hampering the planning and delivery of FP services. Thus, investigating the underlying causes of unmet need for modern contraception in North Gonja is critical for improving the health of mothers and babies in the district. The aim of the present study was therefore to determine the prevalence and determinants of unmet need for contraception in North Gonja District, Ghana.

## Methods

### Study design, area and population

The study was a cross-sectional survey conducted in North Gonja District in March to June, 2016. The North Gonja District is located in the western part of Savanna Region and lies within longitude 1051 and 20,581 West and latitude 80,321 and 10,021 North. The district shares boundaries to the West with West Gonja and Wa East districts, to the East with Tolon District, to the North with Mamprugu-Moagduri and Kumbungu districts and to the South with Central Gonja district [16]. The district has a total land mass of about 4845.5sq km, representing about 6.9% of the total land size of the Region. About 75% of the communities in the district are difficult to access due to the poor nature of the road network and heavy flood during rainy season. This normally affects the delivery of health services in the district. The inhabitants of the district are predominantly Gonjas and Tamplumas but people of other ethnic groups such as Dagomba, Mamprusi, Hanga and Ewe can also be found there.

The District is divided into four health sector operational sub-districts namely Bawena, Daboya, Lingbinsi and Mankarigu and has a total of sixty-one (61) communities. The district has an estimated population of 43,547 [16]. The study population consisted of women aged 15–45 years in the district.

### Sample size and sampling

A minimum sample size of 384 was derived using the formula N = [z^2^ p (1-p)] ÷ ME^2^ [17], where z = confidence interval at 95% thus 1.96; p = assumed prevalence of unmet need for contraception in North Gonja District, Ghana, 50%; and ME = margin of error, 0.05. The sample size was rounded up to 386. Convenience sampling was used to select one sub-district (Daboya) out of the 4 sub-districts in the District because of the relative ease of access and 20 communities were also conveniently selected from this sub-district. Probability proportional to size was used to determine the number of women to sample from each of the selected communities. The respondent women were selected using simple random sampling. Four hundred women were approached to get the 386 women to interview giving a response rate of 96.5%. The 14 women not interviewed were not resident in the study area (*n* = 7), did not consent to participate (*n* = 5) and could not be interviewed because of language barrier (*n* = 2).

### Data collection

Data were collected in face-to-face interviews in respondents’ homes by 4 trained community health nurses who were engaged in the provision of reproductive health services at their work places. The questionnaire used had sections on socio-demographic characteristics, reproductive and fertility history, and knowledge and practice of FP (See Additional file 1). The socio-demographic characteristics included age, highest educational status, marital status, occupation, and religious afilliation. Age in years was self-reported and this was categorised into 5-year groups (i.e., < 20 years, 20–24 years); marital status was categorized into married, and single or divorced (i.e., not currently married); and highest educational level ranged from no education to tertiary level education. Ethnicity had two main categories Gonja and Dagomba and all others were grouped together as “others”; and religion had Muslim and “others”. Information collected on reproduction and fertility history of respondents included age at first birth, number of children, previous contraceptive use, whether they would accept help in limiting/spacing childbirth and reasons for non-use of contraceptives. Issues explored on FP knowledge included what FP was and respondents were also asked to name at least one modern contraceptive.

### Study variables

The dependent variable of the study was unmet need for modern contraception and the independent variables were socio-demographic factors, and contraceptive knowledge and practices of the women.

#### Unmet need for limiting childbirth

The proportion of fecund, sexually active women who wish to stop childbearing  completely but are not using any modern contraceptive method.

#### Unmet need for spacing childbirth

The proportion of fecund, sexually active women who want another child after 2 years but are not using any modern contraceptive method.

#### Unmet need for modern contraception

Any woman having unmet need for limiting or spacing childbirth was considered as having unmet need for contraception [6].

Women were considered to be fecund if they were pregnant or postpartum amenorrheic. Non-pregnant and non-amenorrheic women were classified as fecund if they did not meet the criteria for infecundity i.e., they were married for at least 5 years but did not have a birth in the past 5 years or are not currently pregnant and have never used any contraceptive method; or self-reported infecundity, hysterectomy, or menopause or never menstruated or have been postpartum amenorrheic for at least 5 years; or they are not pregnant or amenorrheic and their last menstrual period occurred more than 6 months prior to the survey. All married women were considered to be sexually active and unmarried women were considered to be sexually active if they had a sexual encounter in the month preceding the survey [6].

### Quality control

The interviewers underwent a 2-day training on the contents of the questionnaire and techniques of questionnaire administration. Prior to data collection, the questionnaire was pretested in a community in the North Gonja district that was not part of the study communities and questions that were unambigous or did not elicit the appropriate responses were rephrased.

### Data management and analysis

Data management and analysis were performed using Stata (version 13, Stata Corporation, College Station, Texas, USA). The determinants of unmet for contraception were identified in a two-stage process. Bivariate associations between unmet need for contraception and the characteristics of the women were explored using Chi-square or Fisher’s exact test and those characteristics that were statistically significant were retained and entered into a logistic regression model. In the logistic regression analysis, odds ratios and their 95% confidence intervals were computed to reflect the strength of association between unmet need and its independent determinants. In all analyses, a *p*-value less than 0.05 was considered statistically significant.

### Ethics approval and consent to participate

The protocol for this study (Protocol Number 04–2016) was approved by the Joint Ethics Board of School of Medicine and Health Sciences and School of Allied Health Sciences, University for Development Studies, Tamale. Written informed consent was obtained from the study women and from the parents of subjects less than 16 years old before interviews were conducted. It was made clear to all subjects that participation was voluntary.

## Results

### Socio-demographic data of respondents

Majority of our sample were within 20–24 years age group (Table 1) with an average age of 26.1 (±8.4) years. The majority were married (64.5%) while a greater majority were Muslims (74.1%) and belonged to Gonja (83.7%), which is the predominant tribe in the study area. With respect to occupation and education, most of them (45.9%) were traders and had education up to Junior High School level (37.0%).

**Table 1:** Socio-demographic data of respondents (*n* = 386)

Variable	Frequency	Percent
**Age group (years)**		
< 20	81	21.0
20–24	100	25.9
25–29	70	18.1
30–34	95	24.6
35+	40	10.4
**Religion**		
Muslim	286	74.1
Others	100	25.9
**Educational level**		
None	76	19.7
Primary	22	5.7
Junior High School	143	37.0
Senior High School	112	29.0
Tertiary	33	8.5
**Ethnicity **		
Gonja	323	83.7
Dagomba	42	10.9
Others	21	5.4
**Marital status**		
Married	249	64.5
Single or Divorced	137	35.5
**Occupation**		
Farmer	38	9.8
Trader	177	45.9
Civil/Public Servant	17	4.1
Student	76	19.7
Unemployed	54	14.0
Others	24	6.5

### Contraception knowledge and practice of the women

We collected information on contraception knowledge and practice of women. Our results show that, awareness on FP among our study population was almost universal. A greater majority of the women (95.9%) were aware of FP and had ever used a modern method or did something to prevent pregnancy (54.1%) as depicted in Table 2. Nevertheless, more than half of them (53.4%) had never used a modern FP method and a large proportion of them (73.6%) would accept any help to avoid getting pregnant. On the reasons for lack of use of modern contraceptives, 29.8% were not using because they were not married or because of fertility-related reasons, 20.2% gave method-related reasons, and 15.3% gave reasons such as opposition to use by self or significant others. About half (50.4%) of those who cited unmarried status and fertility reasons attributed non-use of modern contraceptives to not having sex, 62.8% of those who gave method related-reasons attribute non-use to fear of side effects, and 55.9% of those who cited opposition to use reasons attributed non-use to opposition from husband/partner.

**Table 2:** Contraception knowledge and practice of the women

Characteristic	Frequency	Percent
**Awareness on family planning**		
Yes	370	95.9
No	16	4.1
**Had ever used a method or did something to prevent pregnancy**		
Yes	209	54.1
No	177	45.9
**Had ever used a modern family planning method**		
Yes	180	46.6
No	206	53.4
**Would accept any help to avoid getting pregnant**		
Yes	284	73.6
No	102	26.4
**Reasons for not using modern contraceptives**		
**Unmarried status and fertility-related reasons (*n*** **= 115)**		
Not married	22	19.1
Not having sex	58	50.4
Infrequent sex	18	15.7
Menopausal/hysterectomy	8	7.0
Sub-fecund/infecund	5	4.4
Lactational amenorrhea	2	1.7
Breastfeeding	2	1.7
**Method-related reasons (*n*** **= 78)**		
Health concerns	11	14.1
Fear of side effects	49	62.8
Lack of access to contraceptives	2	2.6
Other reasons	16	20.5
**Opposition to use and lack of knowledge reasons (*n*** **= 59)**		
Respondent opposed	6	10.2
Husband/partner opposed	33	55.9
Others opposed	14	23.7
Religious prohibition	5	8.5
Respondent knows no method	1	1.7

### Prevalence and determinants of unmet need for contraception

The overall prevalence of unmet need for contraception in the north Gonja District was 38.9%, with 27.5% having unmet need for limiting and 12.2% unmet need for spacing childbirth. Unadjusted or bivariate analyses revealed that six variables: age (*p* < 0.001), educational status (p < 0.001), occupation (p < 0.001), awareness on FP (*p* = 0.048), experience with pregnancy prevention in the past (p < 0.001) and previous use of modern contraceptives (p < 0.001) were statistically significantly associated with unmet need for contraception (Table 3). Following multivariate logistic regression analysis, age, educational status, awareness on FP and previous use of any form of contraception remained as independent determinants of unmet need for contraception. Compared to women aged 25–29 years, women aged 20–24 years and those aged 30 years and above were about 75% less likely to have unmet need for contraception [Adjusted Odds Ratio (AOR) 0.26; 95% Confidence Interval (CI) 0.11–0.58; *p* = 0.001; AOR 0.25; 95% CI 0.09–0.73; *p* = 0.011] (Table 4). Women with no formal education were five times more likely to have unmet need for contraception compared with those who had education up to tertiary level (AOR 5.06; 95% CI 1.07–24.01; *p* = 0.041). Furthermore, it was found that, women who were unaware of FP were almost four times more likely to have unmet need (AOR 3.93; 95% CI 1.12–13.80; *p* = 0.033). Finally, women who had never used a modern contraception method nor did anything to prevent pregnancy before were about two times more likely to have unmet need for contraception (AOR 1.81; 95% CI 1.09–3.00; *p* = 0.022).

**Table 3:** Association between characteristics of women and unmet need for contraception status

Characteristic	Total	Unmet need for contraception		Test statistics
		No (%)	Yes (%)	
**Age group (years)**				
< 20	81	42 (51.9)	39 (48.1)	X^2^ = 32.286; *p* < 0.001
20–24	100	83 (83.0)	17 (17.0)	
25–29	70	41 (58.6)	29 (41.4)	
30–34	95	44 (46.3)	51 (53.7)	
35+	40	26 (65.0)	14 (35.0)	
**Educational level**				
None	76	32 (42.1)	44 (57.9)	X^2^ = 19.985; *p* = 0.001
Primary	22	17 (77.3)	5 (22.7)	
Junior High School	143	86 (60.1)	57 (39.9)	
Senior High School	112	75 (67.0)	37 (33.0)	
Tertiary	33	26 (78.8)	7 (21.2)	
**Religion**				
Muslim	286	174 (60.8)	112 (39.2)	X^2^ = 0.042; *p* = 0.838
Others	100	62 (62.0)	38 (38.0)	
**Ethnicity**				
Gonja	323	192 (59.4)	131 (40.6)	X^2^ = 3.234; *p* = 0.198
Dagomba	42	31 (73.8)	11 (26.2)	
Others	21	13 (61.9)	8 (38.1)	
**Marital status**				
Married	249	147 (59.0)	102 (41.0)	X^2^ = 1.307; *p* = 0.253
Single or divorced	137	89 (65.0)	48 (35.0)	
**Occupation**				
Farmer	38	14 (36.8)	24 (63.2)	*p* < 0.001
Trader	177	109 (61.6)	68 (38.4)	
Civil/public servant	17	13 (76.5)	4 (23.5)	
Student	76	41 (53.9)	35 (46.1)	
Unemployed	54	46 (85.2)	8 (14.8)	
Others	24	13 (54.2)	11 (45.8)	
**Awareness on family planning**				
Yes	370	230 (62.2)	140 (37.8)	X^2^ = 3.926; *p* = 0.048
No	16	6 (37.5)	10 (62.5)	
**Had ever used a method or did something to prevent pregnancy**				
Yes	209	157 (75.1)	52 (24.9)	X^2^ = 37.491; *p* < 0.001
No	177	79 (44.6)	98 (55.4)	
**Had ever used a family planning method**				
Yes	180	127 (70.6)	53 (29.4)	X^2^ = 12.585; *p* < 0.001
No	206	109 (52.9)	97 (47.1)	
**Would accept any help to avoid getting pregnant**				
Yes	284	179 (63.0)	105 (37.0)	X^2^ = 1.613; *p* = 0.204
No	102	57 (55.9)	45 (44.1)

**Table 4:** Mulitivariate analysis of the determinants of unmet need for contraception

Characteristic	Adjusted Odds Ratio	95% Confidence Interval	***P***-value
**Age group (years)**			
< 20	0.85	0.36–2.02	0.715
20–24	0.26	0.11–0.58	0.001
25–29	1.00		
30–34	1.31	0.66–2.59	0.436
35+	0.25	0.09–0.73	0.011
**Education**			
None	5.06	1.07–24.01	0.041
Primary	2.74	0.44–17.17	0.281
Junior High School	2.20	0.50–9.65	0.295
Senior High School	2.78	0.64–12.01	0.171
Tertiary	1.00		
**Ethnicity**			
Gonja	1.00		
Dagomba	0.45	0.20–1.01	0.054
Others	0.93	0.32–2.70	0.887
**Occupation**			
Farmer	1.24	0.16–9.35	0.834
Trader	0.56	0.09–3.61	0.538
Civil/public servant	1.00		
Student	1.28	0.20–8.05	0.796
Unemployed	0.22	0.03–1.64	0.141
Others	0.79	0.10–6.20	0.823
**Awareness on family planning**			
Yes	1.00		
No	3.93	1.12–13.80	0.033
**Had ever used a method or did something to prevent pregnancy**			
Yes	1.00		
No	1.81	1.09–3.00	0.022

## Discussion

The primary objective of the present study was to estimate the prevalence of unmet need for contraception and identify its associated factors in North Gonja District, Ghana. We found that 38.9% of our study women have overall unmet need for contraception with more women having unmet need for limiting than for spacing childbirth.

Awareness on FP was almost universal, a finding that is supported by the recent Demographic and Health Survey of Ghana and the United Nations report on world contraceptives [10, 18]. The high awareness possibly reflects the outcome of the work done by both Governmental and Non-Governmental Organisations in promoting reproductive health education in the District and the easy accessibility of reproductive health information due to the Community-based Health Planning and Services compounds, a concept employed to bring health services to the door step of the rural poor in Ghana [19, 20].

Despite the high awareness on FP among women in the study, less than half of the population reported ever use of modern contraceptives culminating in a high prevalence of unmet need for contraception (38.9%). Our prevalence is more than both the national and regional averages of 30 and 28% respectively [10] and the average for Sub-Saharan Africa (31.0%) [21]. However, elsewhere in Africa, a few studies reported lower rates of unmet need (e.g., 16.2% in Ethiopia in 2019 [22], but most studies tend to report higher rates i.e., 51.7% in Angola in 2018 [23], 52.4% in Ethiopia in 2011 [24], and 44.8% in Sudan in 2013 [25]. The high prevalence of unmet need in the present study may be attributed to contraceptive security issues such as distribution problems, fear of side effects, unacceptability of contraception, perception of inability to conceive if contraception is stopped, lack of contraceptive information, opposition from husband to contraceptive use, inability to afford contraceptives, and unaddressed myths, rumours and misconceptions about contraception [12, 26–29]. Nevertheless, the high level of unmet need among these women is a cause for concern and reinforces the importance of improving FP programmes in order to promote contraception and prevent unwanted pregnancy and its attendant problems.

Following multivariate analysis, age, education level, awareness on FP and previous use of contraception were identified as the determinants of unmet need for contraception in the study population. With respect to age, we found that unmet need was less prevalent in younger or older women. A possible explanation for this may be perceived reduced risk of pregnancy due to infrequent sexual activity among younger and older women. Younger women may lack comprehensive education on FP, may not have the resources to access FP services or may be hindered by stigma [22] while older women may not be married or in union.

We found that uneducated women were more likely to have unmet need compared to those with tertiary level education. This finding shows that, women with higher educational status are more likely to use FP compared to their counterparts with no education or lower educational status. Our finding is consistent with findings from other studies which reveal that women with higher levels of education have better access to information on modern contraceptives and health facilities than those with no or low levels of education and education mediates improved contraceptive usage [13, 21, 30–35]. This means that the use of modern contraceptives in order to minimize fertility, morbidity and mortality in mothers and children is better appreciated by educated women. Also, these women may have better chances to seek appropriate counselling from health workers concerning adverse effects of contraceptives and how to avoid them [26]. Moreover, access to education could increase job/employment opportunities and therefore income earning opportunities which may translate into improved contraceptive access and hence usage [24]. The role of education in improving maternal and child health cannot be overemphasized. In 1995, the government of Ghana launched free compulsory universal basic education [36] and a capitation grant programme was launched in 2005 in which school fees were abolished and public basic schools were to receive a grant of 3 United States dollars per child per academic year [37]. In 2017, a programme was started to absorb the cost of secondary school and vocational training for all students [38]. These efforts are contributing greatly to girl child education and may help improve maternal and child health.

Women who were unaware of FP and women who never used a contraception method nor did anything to prevent pregnancy previously were more likely to have unmet need for contraception. A possible reason for this finding is lack of information on FP or wrong perception about FP hence the unwillingness to adopt it. Some studies reported that women perceive FP is for only married couples whilst others hold the believe that it causes injury to the womb [39, 40] or makes one barren in future. Thus, FP programmes and educational messages should highlight the benefits of FP and deal with misconceptions women hold concerning it [41].

### Limitation of the study

Information collected relied on recall of events of the women interviewed and may be influenced by recall bias. Younger and unmarried women may under-declare contraceptive use because of the stigma associated with young age or unmarried marital status and sex.

Despite this limitation, this study is of high importance to current research on unmet need for contraception in the district and region and the findings are generalizable to similar districts in Ghana.

## Conclusions

The present study found high prevalence of both awareness on and unmet need for contraception among the study population. Among all the factors studied, age, educational status, awareness on FP and previous use of contraception emerged as determinants of unmet need for contraception in North Gonja District. Educational campaigns to promote contraception should prioritize women of middle age and low educational status. Further studies are needed to understand the low correlation between awareness on and unmet need for contraception.

## Supplementary information

**Additional file 1.** Questionnaire.

## Data Availability

The minimal dataset analysed during the current study can be obtained from the corresponding author upon reasonable request.

## Abbreviations

FP: Family Planning
AOR: Adjusted Odds Ratio
CI: Confidence Interval
