# Supplementary material for: Prevalence and determinants of unmet need for contraception in North Gonja District, Ghana
Source: BMC Womens Health. 2020 Oct 6;20:222. doi: 10.1186/s12905-020-01077-4 (PMC7539416; doi:10.1186/s12905-020-01077-4)
Supplement: Supplementary file 1 — Additional file 1. Questionnaire. [file 12905_2020_1077_MOESM1_ESM.docx]

**ADDITIONAL FILE 1**

**PREVALENCE AND DETERMINANTS OF UNMET NEED FOR CONTRACEPTION IN NORTH GONJA DISTRICT**

**(TARGET GROUP: WOMEN OF REPRODUCTIVE AGE, 15-45 YEARS)**

**IDENTIFICATION**

Questionnaire ID ………………………………

**A. BACKGROUND INFORMATION OF RESPONDENTS**

1.1 Age in years …………………

1.2. Religion

1. Christian [ ]

2. Muslim [ ]

3. Traditional African Religion [ ]

4. Other (specify) ………………………………………………………….

1.3. Ethnicity

1. Gonja [ ]

2. Dagomba [ ]

3. Dagaaba [ ]

4. Other (specify) ………………………………......................................

1.4 Marital Status

1. Single [ ]

2. Married [ ]

3. Divorced [ ]

4. Other (specify) ………………….........................................................

1.5 Occupation

1. Farmer [ ]

2. Trader [ ]

3. Unemployed [ ]

4. Civil/public servant [ ]

5. Student [ ]

6. Other (specify) …………………………………………………………

1.6 Level of education

1. Primary [ ]

2. Middle/Junior High School [ ]

3. Senior High School [ ]

4. Tertiary [ ]

5. None [ ]

**B. KNOWLEDGE ON AND PRACTICE OF CONTRACEPTION**

2.1 Have you ever heard of family planning?

1. Yes [ ]

2. No [ ] [*If response to Question 2.1 is 2. No, skip to Section C*]

2.2 Where did you hear of family planning?

1. Media [ ]

2. Relatives [ ]

3. Friends [ ]

4. NGOs [ ]

5. Other, specify ……………………………………………………………………

2.3 What is family planning? .........................................................................................................................

2.4 Mention any two types of family planning methods you know.

1.…………………………………………………………………………………………… 2…………………………………………………………………………………………….

2.5 Have you ever used any family planning method?

1. Yes [ ]

2. No [ ]

*[If response to Question 2.5 is 2. No, skip to Section C.]*

2.5 If yes, which family planning method(s) did you use? ………………………………............................................................................

2.6 Why did you choose to use the family planning method(s) mentioned in Question 2.5? .......................................................................................................................

**C REPRODUCTION HISTORY AND LEVEL OF UNMET NEED FOR CONTRACEPTION**

I will like to ask you about any children you have had. I am interested in all your biological children.

3.1. Have you ever been pregnant? *[Include current pregnancy if pregnant]*

1. Yes [ ]

2. No [ ]

3. NA [ ]

*[Skip to Question 3.11 if response to Question 3.1 is 2. No]*

3.2. How many times have you ever been pregnant? *[Include current pregnancy if pregnant]* …..........................................................................................................................................

3.3 Have you ever given birth?

1. Yes [ ]

2. No [ ]

3. NA [ ] *[If response to Question 3.3 is not 1 Yes, skip to Question 3.6]*

3.4 How old were you when you first gave birth? [In years] ………………………..

3.5 How many children have you given birth to? ………………

3.6 Women sometime have pregnancies that do not result in a live birth, that is a pregnancy can end early in a miscarriage, abortion or the baby can be born dead (Stillbirth).

Have you had any such pregnancy that did not result in a live birth?

1. Yes [ ]

2. No [ ]

3. NA [ ]

3.7 In all, how many pregnancies did you have that did not end in a live birth? ………….

3.8 Are you pregnant now?

1. Yes [ ]

2. No [ ]

3. NK [ ] *[If answer to question 3.8 is not 1 Yes, skip to Question 3.11]*

3.9. How many months pregnant are you? [Record number of completed months. Refer to ANC card if available] …………………..

3.10 Thinking to back the time when your current pregnancy started, would you say that you wanted the pregnancy then, later, or not at all?

1. Then [ ]

2. Later [ ]

3. Not at all [ ]

4. NK [ ]

5. NA [ ] *[Skip Question 3.10.1 if response to Question 3.10 is not 2. Later]*

3.10.1. How much longer would you have liked to wait in months? ……………..

3.11 Sometimes a woman can become pregnant when she is not ready. In the past, have you ever been pregnant at a time when you were not ready for the pregnancy?

1. Yes [ ]

2. No [ ]

3. NK [ ]

4. NA [ ] *[Answer Questions 3.12 to 3.14 if the response to Question 3.11 is 1 Yes]*

3.12 How many pregnancies have you had when you were not ready? …………………….

3.13 What did you do the last time you had a pregnancy that you were not ready for?

1. Nothing, continued with the pregnancy

2. Attempted to stop but did not succeed

3. Attempted to stop and succeeded

4. Other, specify ………………………………………………

5. NK

6. NA

3.14 How old were you the last time you had a pregnancy that you were not ready for? [age in completed years]…………………………………………..

3.15 Would you accept any help to prevent you from becoming pregnant?

1. Yes [ ]

2. No [ ]

3. NA [ ]

3.16 Have you ever used anything or tried in any way to delay or avoid getting pregnant?

1. Yes [ ]

2. No [ ]

3. NA [ ]

*[Ask Question 3.17 if the answer to Question 3.16 is 1. Yes, otherwise skip to Question 3.18]*

3.17 What have you used or done to delay or avoid getting pregnant? .............................................................................................

3.18 Are you currently doing something or using any method to delay or avoid getting pregnant?

1. Yes [ ]

2. No [ ]

3. NA [ ] *[If the respond to Question 3.18 is not 1. Yes, skip to Section E]*

**D. CHOICE OF CONTRACEPTIVE**

4.1. Which contraceptives are you currently using?

| CONTRACEPTIVE | TICK CONTRACEPTIVE(S) BEING USED |
| --- | --- |
| 1. Female sterilization |  |
| 2. IUD |  |
| 3. Male condom |  |
| 4. Foam/Jelly |  |
| 5.Withdrawal |  |
| 6. Male sterilization |  |
| 7. Injectable |  |
| 8. Female condom |  |
| 9. Lactational amenorrhea method |  |
| 10. Pill |  |
| 11. Implant |  |
| 12. Diaphragm |  |
| 13. Rhythm Method |  |

*[Ask Question 4.2 if the responses to Question 4.1 include any of the following: 2. IUD, 3. Male condom, 4. Foam/Jelly, 7. Injectable, 8. Female condom, 10. Pill, 11. Implant,*

*12. Diaphragm]*

4.2. Since what month and year have you started using the (CURRENT CONTRACEPTIVE) without stopping? [mm, yyyy] …………………………..

4.3. Does your husband/partner know that you are using a contraceptive?

1. Yes [ ]

2. No [ ]

3. NK [ ]

4. NA [ ]

*[Ask Question 4.4 if the answer to Question 4.3 is 1. Yes]*

4.4. Would you say that using of contraceptive is mainly your decision, mainly your husband’s/partner’s decision, or did you both decide?

1. Mainly respondent

2. Mainly husband/partner

3. Joint decision

4. Other

5. NK

6. NA

4.5. When was the last time you had sexual intercourse?

1. Days ago…………………..

2. Weeks ago………………….

3. Months ago…………………

4. Years ago……………………

**E FERTILITY PREFERENCES**

I will now like to speak to you about your current pregnancy (if pregnant) and your future childbearing intentions.

5.1. Are you currently pregnant?

1. Yes [ ]

2. No [ ]

3. NK [ ]

4. NA [ ]

*[Go to Questions 5.4 if the response to Question 5.1 is not 1. Yes. ]*

5.2. After the child you are expecting now, would you like to have another child, or would you prefer not to have any more children?

1. Have another child

2. Prefer not to have a child or no more children

3. NK or Undecided

4. NA

*[If the answer to Question 5.2 is not 1. Have another child, skip to Question 5.4]*

5.3. After the birth of the child you are expecting now, how long would you like to wait before the birth of another child?

1 …………………… Months

2. NK

3. NA

*[Now I have some questions about the future.]*

5.4. Would you like to have a child or another child, or would you prefer not to have any child or more children?

1. Have a child or another child

2. Prefer not to have a child or no more children

3. Can’t get pregnant

4. NK or Undecided

5. NA

*[Ask question 5.5 if response to question 5.4 is 1. Have a child or another child]*

5.5. How long would you like to wait from now before the birth of a child or another child?

1. Months/years

2. Want a child soon or now

3. After Marriage

4. Can’t get pregnant

5. Other

7. NK

8. NA

5.6. If question 5.5 is 1. Months/years, how many months or years? ………………

*[Check if the woman is using any method of contraception. If she is not using any method of contraception and does not want a child soon or does not want a child altogether ask Question 5.7]*

5.7. You have said that you do not want a child or another child soon or you do not want a child altogether, but you are not using any contraceptive to avoid pregnancy. Can you tell me why you are not using any contraceptive?

| REASONS FOR NOT USING CONTRACEPTIVE | TICK IF CONTRACEPTIVE IS USED |
| --- | --- |
| MARITAL AND FERTILITY-RELATED REASONS |  |
| 1. Not married |  |
| 2. Not having sex |  |
| 3. Infrequent sex |  |
| 4. Menopausal/hysterectomy |  |
| 5. Sub-fecund/infecund |  |
| 6. Lactational amenorrhea |  |
| 7. Breast feeding |  |
| OPPOSITION TO USE AND LACK OF KNOWLEDGE REASONS |  |
| 8. Respondent opposed |  |
| 9. Husband/partner opposed |  |
| 10. Others opposed |  |
| 11. Religious prohibition |  |
| 12. Knows no method |  |
| 13. Knows no source |  |
| METHOD-RELATED REASONS |  |
| 14. Health concerns |  |
| 15. Fear of side effects |  |
| 16. Lack of access/too far |  |
| 17. Cost too much |  |
| 18. Interferes with body |  |
| 19. Other (specify) ………………………………………… |  |

5.8. Do you think you will use a contraceptive to delay or avoid pregnancy in the future?

1. Yes [ ]

2. No [ ]

3. NK [ ] *[Ask Question 5.9 if response to Question 5.8 is 2. No]*

5.9. Why do you think you will not use a contraceptive in the future? .....................................

5.10. Are you currently married, living together with a man, widowed, divorced, separated or never married?

1. Married

2. Living together with a man

3. Widowed

4. Divorced

5. Separated

6. Never married

*[Ask Question 5.11 if the response to Question 5.10 is 1. Married or 2. Living together with a man]*

5.11. How old were you when you first married/lived together with a man? [In years] ……
